# Automatic exudate and aneurysm segmentation in OCT images using UNET++ and hyperreflective-foci feature based bagged tree ensemble

**DOI:** 10.1371/journal.pone.0304146

**Published:** 2024-05-24

**Authors:** Rinrada Tanthanathewin, Warissaporn Wongrattanapipat, Tin Tin Khaing, Pakinee Aimmanee

**Affiliations:** School of Information, Computer and Communication Technology, Sirindhorn International Institute of Technology, Thammasat University, Meung, Patumthani, Thailand; Islamia University of Bahawalpur: The Islamia University of Bahawalpur Pakistan, PAKISTAN

## Abstract

Diabetic retinopathy’s signs, such as exudates (EXs) and aneurysms (ANs), initially develop from under the retinal surface detectable from optical coherence tomography (OCT) images. Detecting these signs helps ophthalmologists diagnose DR sooner. Detecting and segmenting exudates (EXs) and aneurysms (ANs) in medical images is challenging due to their small size, similarity to other hyperreflective regions, noise presence, and low background contrast. Furthermore, the scarcity of public OCT images featuring these abnormalities has limited the number of studies related to the automatic segmentation of EXs and ANs, and the reported performance of such studies has not been satisfactory. This work proposes an efficient algorithm that can automatically segment these anomalies by improving key steps in the process. The potential area where these hyper-reflective EXs and ANs occur was scoped by our method using a deep-learning U-Net++ program. From this area, the candidates for EX-AN were segmented using the adaptive thresholding method. Nine features based on appearances, locations, and shadow markers were extracted from these candidates. They were trained and tested using bagged tree ensemble classifiers to obtain only EX-AN blobs. The proposed method was tested on a collection of a public dataset comprising 80 images with hand-drawn ground truths. The experimental results showed that our method could segment EX-AN blobs with average recall, precision, and F1-measure as 87.9%, 86.1%, and 87.0%, respectively. Its F1-measure drastically outperformed two comparative methods, binary thresholding and watershed (BT-WS) and adaptive thresholding with shadow tracking (AT-ST), by 78.0% and 82.1%, respectively.

## Introduction

**Fig 1:**
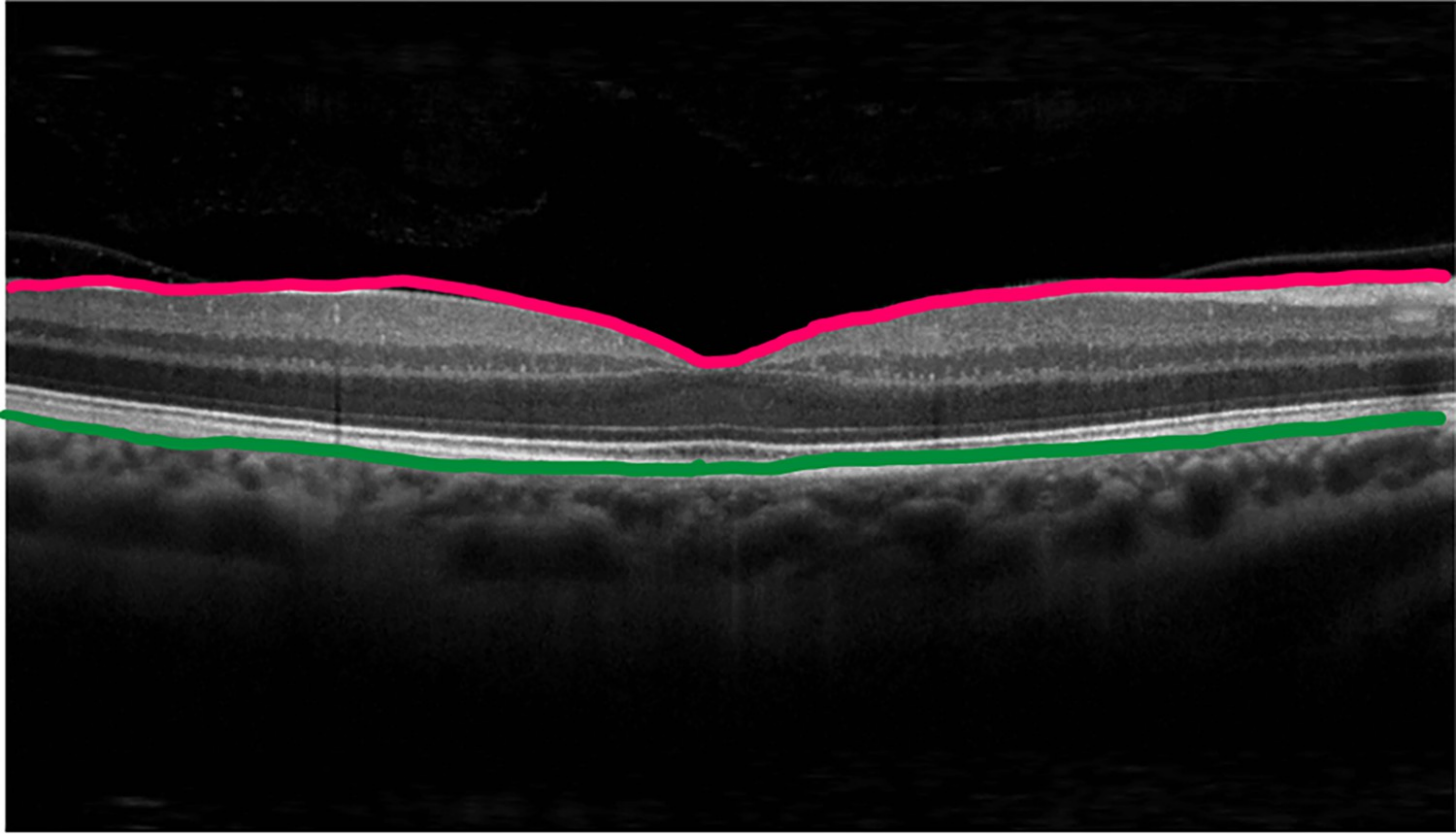
ILM (pink line) and RPE (green line) layers in a normal OCT image.

Optical Coherence Tomography (OCT) is a non-invasive and micron-scale imaging method that uses infrared light waves for visualizing cross-sectional views of morphological structures of tissues [1]. OCT technology is primarily applied for imaging the anterior and posterior segments of the human eye, including the retinal anatomy, the macula, and the optic disc’s depth [2, 3]. In typical OCT images, when oriented upward, the retinal layers exhibit symmetrical y-axis patterns stacked on one another. Fig 1 illustrates the OCT image and tissue layers in a normal case, typically presented in grayscale. The uppermost layer, resembling a bird wing, is known as the internal limiting membrane (ILM), with its central depression referred to as the macula. Another crucial layer is the retinal pigment epithelium (RPE), depicted as a slender line beneath the image’s brightest band. The RPE plays a vital role in supporting and nourishing photoreceptor cells, and its distinctive appearance in imaging assesses retinal health and identifies abnormalities. Beneath the RPE band is a substantial, cloud-like layer known as the choroid. This vascular layer supplies oxygen and nutrition to support the retina. Under normal circumstances, nine flat and smooth layers were typically found between the ILM and RPE lines. These layers manifest in varying shades of gray in the image, and there are no discernible bright spots. Detailed information about OCT images and other layers can be found in the studies of Huang et al., Swanson et al., and Hee et al. [1–3].

OCT imaging has demonstrated great potential and impact in ophthalmology for diagnosing and prescreening ocular diseases [4] because it provides quantitative information on retinal pathology that helps diagnose potential diseases. One of the diseases in which structural abnormalities are detectable within a patient’s retina is diabetic retinopathy (DR) [5]. DR is one of the leading causes of vision loss and blindness in adults between 20-79 years. In 2021, 537 million adults worldwide were living with diabetes [6], and 6.7 million deaths occurred due to diabetes [7]. The global prevalence of the DR population was predicted to rise to 643 million by 2030 and 783 million by 2045 [8]. These unfortunate scenarios can be preventable if DR signs are detected before physical symptoms appear and the patients receive proper treatments to control risk causes such as blood pressure and cholesterols.

**Fig 2:**
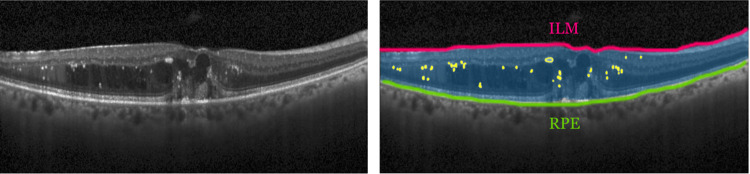
An example of an OCT image of a DR case (left) and illustration of relevant parts (right): EX-AN blobs (yellow blobs) in the area of interest (a blue region), the ILM (a red line) and RPE (a green line) layers.

The DR anomalies, such as hemorrhages (HMs), exudates (EXs), and Aneurysms (ANs), can be seen in both the retinal fundus and OCT images. As these signs build up under the retinal surface before emerging at the top, monitoring them early in the OCT images helps prescreen DR more effectively than in the retinal fundus images. These anomalies are commonly observed as blobs. The HM blobs are caused by bleeding from a damaged blood vessel. They typically occur within the outer nuclear layer and the inner plexiform layer [9]. The EXs are caused by lipid and proteinaceous materials, such as fibrinogen and albumin, leaking from the vessels. The ANs bulge in a blood vessel because of a fragile wall. The EX and AN blobs appear similarly as hyperreflective dots or spots, so-called Hyperreflective Foci (HRF) arising between the internal limiting membrane (ILM) and the retinal pigment epithelium (RPE) [10]. The significance of hyperreflective blobs has been analyzed as the biomarkers in OCT images of several retinal diseases, including DR [11]. In this work, EXs and ANs are considered inseparably. For simplicity, throughout this paper, we call a blob that can be either an exudate or an aneurysm an EX-AN. When the EX-AN blobs are detected in an OCT image of a patient, it strongly indicates that the person is DR. Fig 2 shows EX-AN blobs between the ILM and RPE layers in an OCT image.

This research is crucial for advancing the development of precise diagnostic assistance systems tailored for segmenting exudates and aneurysms during DR prescreening using OCT images. These systems play an important role in supporting ophthalmologists, offering valuable assistance in decision-making throughout the diagnostic process. Beyond its application in ophthalmology, the findings from this work can be extended to other domains, such as histopathology image analysis, cancer detection and grading, and infectious disease diagnosis.

The main contributions of our work are as follows:

- Using physical and pathological observations of EXs and ANs as features for classification hasn’t been explored fully in any other work.
- We are integrating deep learning and machine learning to obtain optimal feasible solutions for each step in the algorithm.
- Our proposed algorithm was evaluated qualitatively and quantitatively and compared against the state-of-the-art methods.

The paper is structured as follows. The Related Work section presents relevant information on prior research. The Methodology section outlines the procedures and techniques employed. In the Datasets and Evaluation Schemes section, we detail the datasets used and our methods for evaluation. The Experiments and Results section elucidates the experimental process and presents findings along with a summary. Subsequently, the Discussion section engages in analysis and interpretation of the results. Finally, the Conclusion section encapsulates the essence of our work.

## Related work

The reviews were classified into tasks: ILM and RPE detection for defining the area of interest, hyperreflective blob segmentation, and exudate and hemorrhage blob segmentation. We grouped reviews by the main techniques for each task: image processing, machine learning, and deep learning.

### ILM and RPE detection

A summary of work related to ILM and RPE detection is as follows. Mokhtari et al. [12] automatically initialized an area of interest between ILM and RPE using the ridgelet transform. They applied an automatic HRF detection method in OCT images with diabetic macular edema using morphological component analysis based on wavelet and curvelet dictionaries constructions. Chen et al. [13] segmented the ILM, RPE, and Bruch’s Membrane with age-related macular degeneration in OCT images. They used a deep forest prediction model integrated with graph theory and dynamic programming. Dodo et al. [14] segmented five distinct layers between the ILM and RPE layers by applying fuzzy C-means and graph-cut methods to handle image inhomogeneity. Okuwobi et al. [15, 16] employed a random forest classifier for probability calculations and an optimal graph search method. Subsequently, they applied the Sobel edge algorithm to identify the Inner Limiting Membrane (ILM) and Retinal Pigment Epithelium (RPE) boundary, defining this region as the area of interest.

It is worth noting that most traditional image processing algorithms usually require post-processing steps to produce smooth boundaries of retinal layers. In addition, the graph-based model was generally susceptible to the image quality and regularity of the OCT images. It usually worked fine when the quality was good and contained no signs of diseases. Retinal pathologies can lead to deterioration and deformation of the retinal layers, further complicating the segmentation process.

Deep learning has become a promising advancement that offers automatic robust retinal layer segmentation [17, 18]. Mukherjee et al. [17] proposed a three-dimensional deep neural network to learn the ILM, RPE, and Bruch’s Membrane boundaries in OCT volumes with age-related macular degeneration. In recent years, U-Net [19] and its variants, for example, U-Net++ and U-Net++ refinement, have been widely used to perform image segmentation in all medical imaging modalities, such as ultrasound, CT, magnetic resonance imaging (MRI), and OCT images in various applications [20–22]. Kugelman et al. [23] compared the retinal layer segmentation performances of U-Net and its seven variants (U-Net++, Attention U-Net, Dense U-Net, Inception U-Net, R2 U-Net, Residual U-Net, and SE U-Net) using one healthy, two diseased, and one widefield OCT datasets. They concluded the baseline U-Net serves as the most preferred option for retinal layer segmentation in OCT images, considering factors such as training and evaluation time and its simpler complexity despite a slight decrease in performance. Yojana and Thillai Rani [24] employed a hybrid U-Net model with a ResNet34 encoder to segment the retinal layers in OCT images with DR. In their evaluation, the proposed model was compared with U-Net++ and DeepLabV3+. The results indicated their proposed model’s superior performance, followed by the second-best U-Net++ and then DeepLabV3+.

### HRF segmentation

For the quantification of HRF areas in SD-OCT images, Okuwobi et al. [16] segmented HRF using morphological image reconstruction and histogram information and extracting the extreme value area from the connected region of the component tree. An F1-measure score of 70.1% was reported. Their proposed work outperformed the previous grow-cut method [15] by 9.7% and the fully convolutional network [25] by 9.9%, respectively. The traditional image processing algorithms showed more satisfactory segmentation accuracy of HRF over convolutional networks. One limitation of the morphological technique is sensitivity to variations in pixel intensities.

Methods of a deep learning approach were popularly selected for HRF blob classification, detection, and segmentation in OCT images. Katona et al. [26] used deep neural networks to segment and quantify HRF blobs and age-related macular degeneration biomarkers in retinal OCT images. Schlegl et al. [27] proposed a U-Net model that integrates a residual module for segmenting HRF blobs in OCT images. They achieved the best recall, precision, and F1-measure of 76.88%, 66.55%, and 71.34%, respectively. For automatic HRF segmentation in SD-OCT images, Yu et al. [28] constructed a model based on deep classification networks using GoogLeNet and ResNet. Small lesions were segmented through pixel-wise predictions in small patches. They obtained an F1-measure score of 67.8% for HRF segmentation in the cropped foci area in OCT B-scans. Varga et al. [29] tested the HRF segmentation performances of several existing networks on two small datasets. The methods tested were deep rectifier neural networks, fully convolutional neural networks, and image processing. They reported that neural networks yielded the maximum F1-measure score of over 80%. To segment HRF areas in SD-OCT volumes, Xie et al. [30] presented a three-dimensional U-Net model comprising three slice-wise dilated convolutions. Their technique was done in the bottleneck layer of the network after applying image enhancement and denoising. They obtained an F1-measure score of 70.7% on 33 SD-OCT volumes from DR patients. Yao et al. [31] developed a global information fusion and dual decoder collaboration-based network (GD-Net). It could aggregate global semantic information effectively and learn the semantic correlation between multi-class HRF that contained hard EXs and microglia. The multi-class HRF segmentation performance of GD-Net was tested on 202 OCT B-scan images with diabetic macular edema. It showed an F1-measure score of 62.8%. Wei et al. [32] proposed a lightweight network consisting of two main processes: pre-processing and automatic segmentation of HRF. The non-local mean (NLM) filter and patch-based split techniques were employed in the pre-processing step. The HRF segmentation was performed using a lightweight neural network comprising a dilated convolution layer, batch normalization layer, and ReLU activation function, so-called DBR blocks. The HRF segmentation performance measured by dice similarity coefficient was up to 83.65% from experimenting on 3,000 OCT images of 300 patients with macular edema, retina vein occlusion, and central serous chorioretinopathy. Schmidt et al. [33] used a blob detector based on an image analysis algorithm to detect candidate foci. They used a convolutional neural network (CNN) to select foci in the outer nuclear layer. The test was done on 2,596 OCT B-scans from fourteen eyes of seven patients. They achieved an accuracy of 89%. However, deep learning approaches can be computationally intensive and often require substantial amounts of labeled training data to perform effectively. Additionally, their black-box nature may limit interpretability.

### Exudates and aneurysm segmentation

Niu et al. [34] presented a multimodality analysis based on both retinal fundus and OCT images to investigate the correlations between HRF and EXs and predict DR severity. They used a saliency method to segment hard exudate regions from the cropped images at different scales. However, their segmentation performance was poor, and manual correction was required as post-processing. The SD-OCT en face and cropped fundus images were semi-automatically registered and aligned according to the segmented EX blobs.

Szymkowski et al. [35] detected EXs using the thresholding technique to find hyperreflective areas on color SD-OCT images produced by a Heidelberg spectralis pseudo-color OCT machine. The reported accuracy was as high as 97%. We noticed that in OCT images generated by this particular machine, the Exs stood out from the background, making them easier to detect than other types of OCT images.

Patil and Chakravorty [36] proposed a detection method of EXs in DR’s visual deficiency through OCT images. Pre-processing was first applied to the OCT images, followed by intensity thresholding to separate foreground and background. Watershed segmentation was used to combine nearby pixels into a basin. Edge segmentation was then applied to separate basins to get EX blobs. We discovered that the algorithm was unsuitable for EXs segmentation, primarily because of its well-known drawback of being sensitive to noise. Consequently, the method resulted in over-segmentation.

Singh et al. [37] also proposed a method to localize EX blobs by analyzing their shadowing effect. They used only three images from different SD-OCT instruments and calculated the summation of normalized pixel intensity across the image between the ILM and RPE layers. The differentiation of the intensity-summation curve was analyzed to detect the areas of shadows, which are assumed to be the location of EXs. They provided no numerical results but only concluded that the results were consistent with the diagnostic opinion of the ophthalmologist. Their experiment faced significant drawbacks. Firstly, the number of tested images was insufficient. Secondly, detecting the shadow area relied on the blob’s intensity against the entire image intensity, proving ineffective, particularly in the case of diseased OCT images.

Midena et al. [38] proposed a semi-automatic method for HRF detection in OCT images using ImageJ software with four different settings profiles. They experimented on OCT images of DR patients and obtained a high intraclass correlation value between the ground truth and each of the four semi-automated methods. The intraclass correlation scores reported were between 0.92-0.98. The limitation of this work is that it was not fully automatic and did not evaluate using standard segmentation assessment.

OCT imaging has become widely used in ophthalmology in less than 30 years, but most literature focuses on its clinical applications rather than computerized analysis. Consequently, few studies and available datasets exist for the automatic detection and segmentation of EX-ANs in OCT images. This research addresses this gap by presenting an algorithm that can automatically segment EX-ANs in OCT images. However, there were several challenges to this approach. Firstly, EX-ANs are relatively small compared to the overall image. Secondly, the colorless spectral domain OCT (SD-OCT) images used in our experiment make it more challenging to differentiate EX-ANs from HRFs. Thirdly, the contrast of EX-ANs with the background is low, and other areas in the image can be just as bright. Despite these challenges, our algorithm achieved the best performance in experimental results.

## Methodology

**Fig 3:**
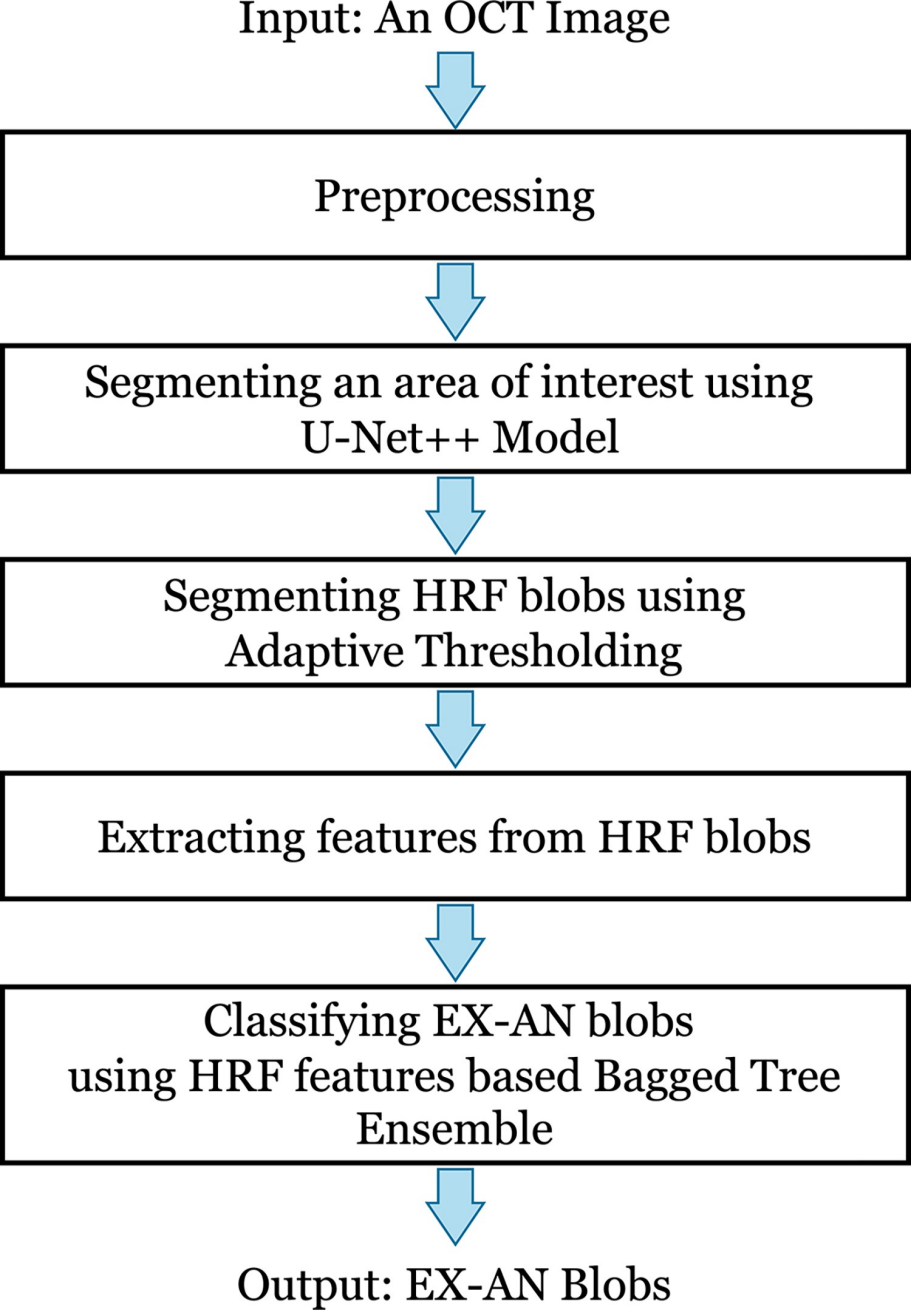
The flowchart depicting the overall procedures of the proposed work.

The overall framework of the proposed method is illustrated in Fig 3. Each process is described in more detail in the following sub-sections.

### Pre-processing

**Fig 4:**
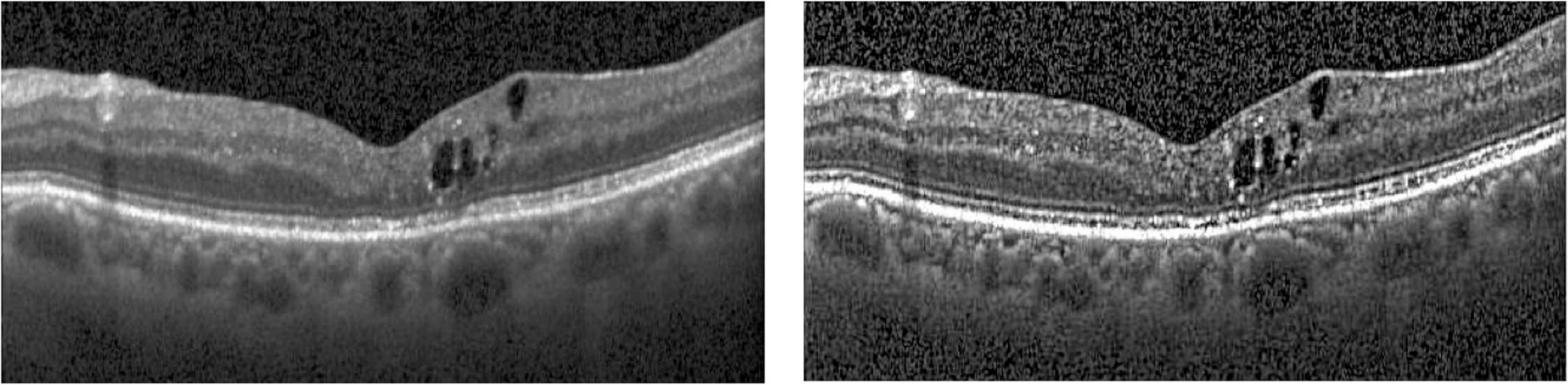
Comparison of images before and after pre-processing: the original image (left) and after applying top-hat and bottom-hat filters (right).

The point intensity in the image was normalized between 0 (black) and 1 (white) by dividing each pixel intensity by the image’s maximum intensity value. The contrast was adjusted by applying top-hat and bottom-hat filtering to detect hyperreflective regions better. The top-hat filter highlighted the bright areas from the dark background, while bottom-hat filtering enhanced the contrast of the dark areas from the bright background. We utilized these filters in our research because experimental evidence demonstrated their superior efficacy in extracting small elements such as EXANs, surpassing the performance of alternative filters. Fig 4 shows images before and after pre-processing.

### Segmenting the area of interest using deep learning UNET++ model

As EX-ANs only presented between the ILM and RPE layers, our program used the ILM and RPE layers as the boundary of our area of interest. These layers of the image were segmented using a deep-learning U-Net++ model. U-Net++ [19] is an enhanced variant of the U-Net architecture [16] that contains densely connected nested sub-networks and skip connections. U-Net++ was experimentally proven less computationally expensive than other U-Net models, yet achieving a comparable high F1 score [15]. Therefore, it was the chosen model for our study.

During the training process of U-Net++, patch images were used. Various data augmentation techniques were incorporated, such as affine transformation, horizontal flipping, random distortion, and zooming. In configuring parameters for this process, we applied a 25-degree rotation angle for the affine transformation and utilized a zoom range between 0.5 and 1.2. Additionally, the random distortion involved a 3×3 grid with a magnitude randomly selected from one to eight, enhancing the variability in the transformations. It is important to note that image transformations using augmentation techniques can generally alter the spatial arrangement of objects within an image. However, in our specific case, these transformations were employed to detect RPE and ILM lines, which serve as edges defining the area of interest (ROI). EX-ANs were segmented within the ROI scope on the original image using a machine-learning approach based on hyperreflective-foci features. Therefore, the locations of EX-ANs would not be impacted by these data augmentation techniques.

**Fig 5:**
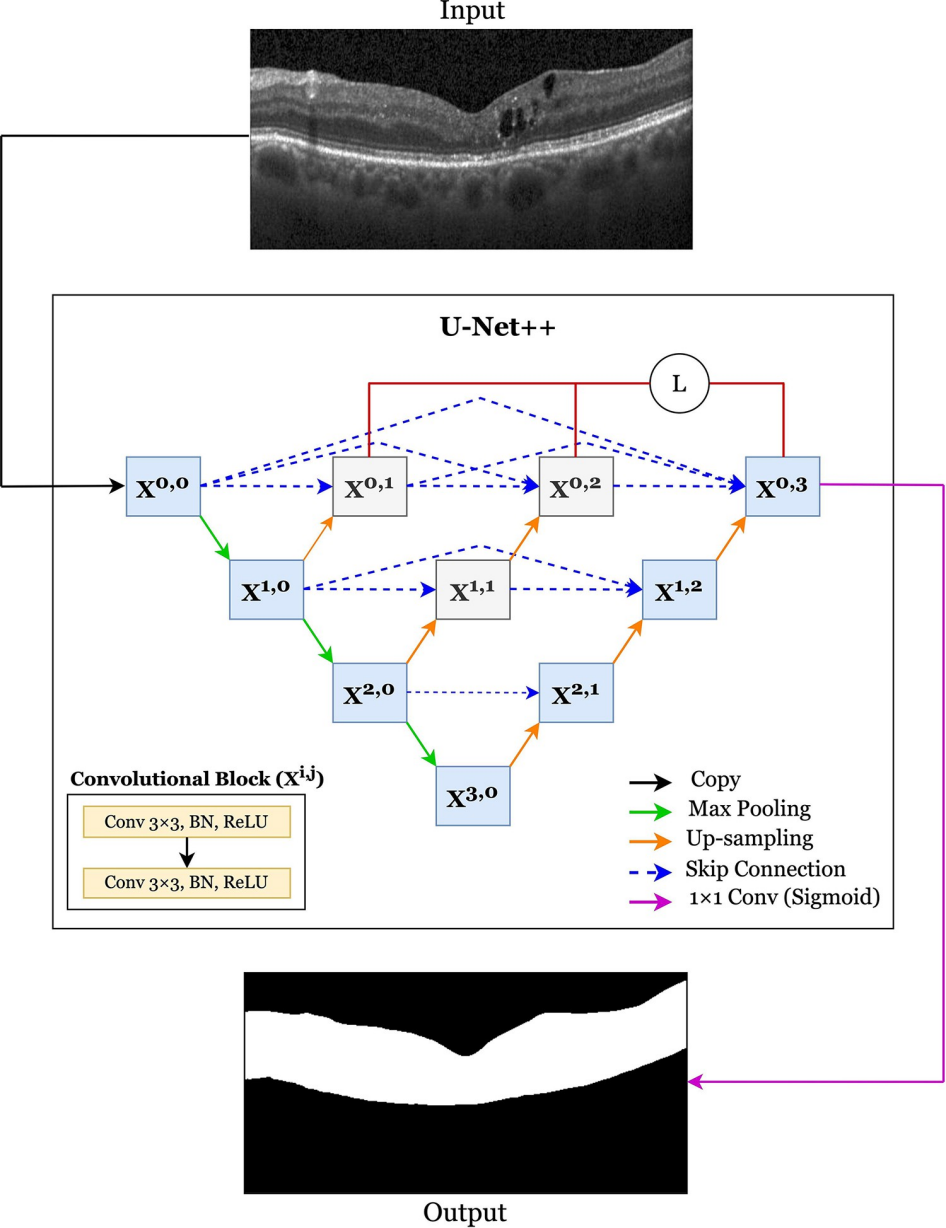
An overview of the U-Net++ architecture for the area of interest selection.

The architecture of U-Net++ is illustrated in Fig 5. A convolutional block had two consecutive 3×3 standard convolutions. It produced an activation map (*X^i,j^*) where *i* denotes the max-pooling layer along the encoder, and *j* indicates the up-sampling layer along the skip pathways. All layers in a convolutional block were batch-normalized (BN) and activated by a rectified linear unit (ReLU). Similar to U-Net, the number of feature vectors was doubled along the encoder and was halved along the decoder.

A 1×1 convolution and a sigmoid activation were applied at the output layer of the network. The sigmoid activation predicted the output of the last node (X^0,3^) into a probability map, where each pixel value corresponded to the likelihood of belonging to a specific class. Pixels with probabilities greater than a certain threshold were identified as belonging to the region of interest, which was a region located between the ILM and RPE layers. Conversely, pixels with probabilities lower than the threshold were classified outside the area.

In a conventional U-net, the activation maps of the encoder path were directly concatenated with the corresponding up-sampled maps in the decoder path through skip connections. The gray convolutional blocks distinguished U-Net++ from U-Net. Using nested skip pathways aided U-Net++ in bridging the semantic gaps and flowing the gradient information between the encoder and decoder paths. This bridging enabled it to perform segmentation more precisely.

### Segmenting HRF blobs using adaptive thresholding

All HRF blobs were used as candidates for the EX-AN blobs. Our program detected hyperreflective blobs within the segmented area of interest. The adaptive thresholding (AT) method based on an intensity histogram was used to obtain bright blobs to produce candidates of EX-AN. This thresholding technique was used because of its simplicity and computational efficiency for many pixels with varying illumination.

The threshold value (*T*) follows Eq (1) for each pixel’s intensity I_ij_.

$$T(I_{ij},Img)=\left\{ \begin{array}{c} 1\;I_{ij}\leq \tau (Img)\\ 0,\;I_{ij}>\tau (Img) \end{array}\right.$$ (1)

where *τ*(*Img*) is the threshold value for an input image *Img*.

We analyzed the optimal threshold value for filtering out non-EX-AN HRF blobs while retaining most EX-AN blobs. We examined the normalized intensity histogram’s tail threshold values of 0.9, 0.8, 0.7, 0.6, and 0.5 from a subset of images. To evaluate the effectiveness of the threshold, we used the ratio of the number of EX-ANs to the number of blobs with normalized intensity above the threshold as an objective function. Based on our analysis, a threshold value of 0.7 provided the highest ratio.

**Fig 6:**
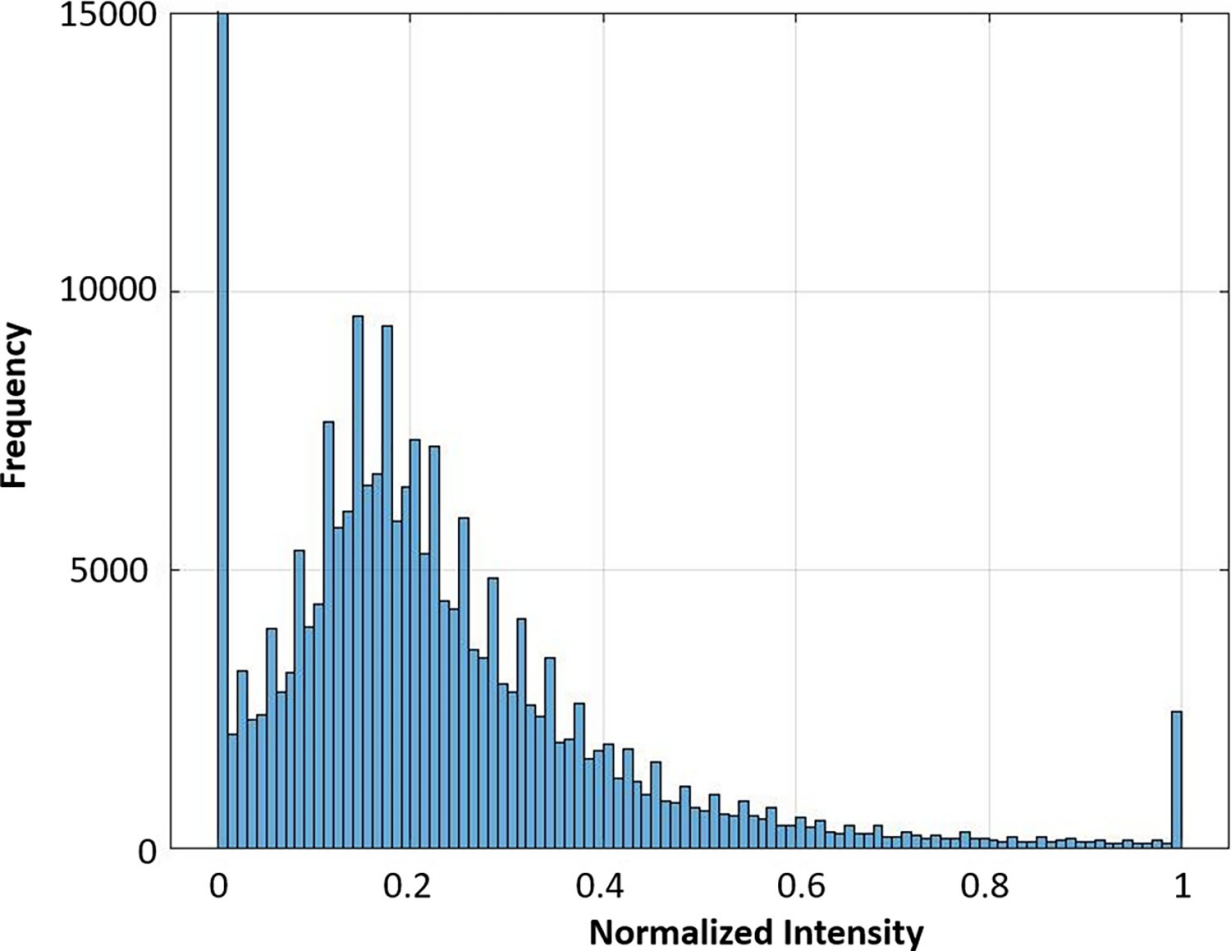
An example of a histogram of the normalized intensity of an OCT image.

Fig 6 shows an example of a histogram of the normalized intensity of an OCT image.

**Fig 7:**
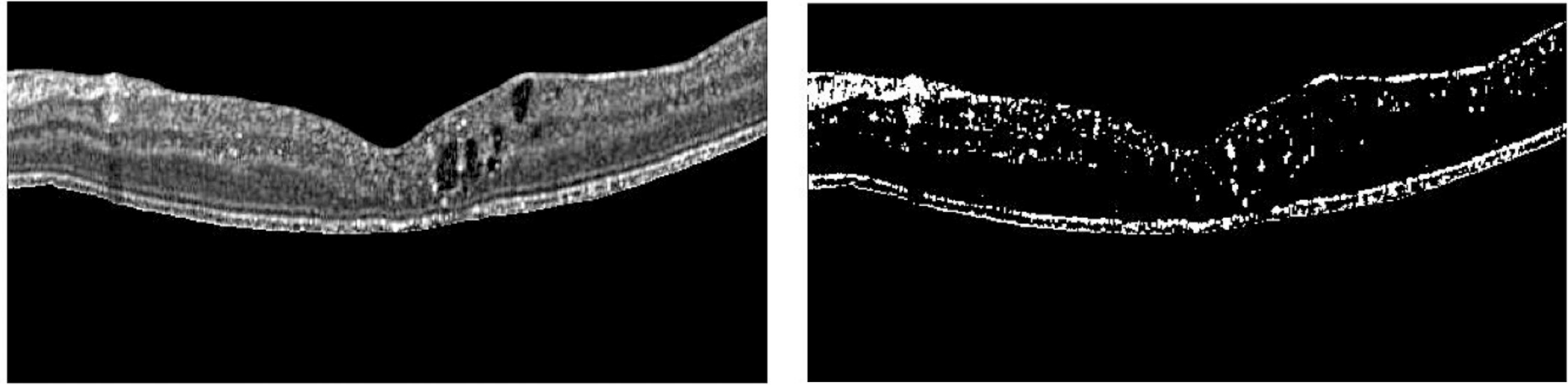
Illustration of Area of interest (left) and all HRF blobs used as EX-AN candidates (right).

Fig 7 shows the HRF blobs obtained after applying an adaptive thresholding method on the region of interest. These blobs were used as the candidates of the EX-ANs.

### Extracting features from HRF blobs

The following nine features were extracted based on the appearance, location, and black shadow markers of candidate blobs obtained from a prior step.

1. The average intensity
2. The minimum intensity
3. The maximum intensity
4. The blob’s size is measured by the number of points in the blob.
5. The distance to fovea
6. The distance to the ILM line
7. The distance to the RPE line
8. The absolute average intensity difference between the focused area beneath the blob and its left area
9. The absolute average intensity difference between the concentrated area beneath the blob and its right area

The observations, definitions, and importance of these features are explained by categories as follows:

#### Appearances

The EX-ANs were highly reflective and always appeared as bright blobs in the OCT images. Thus, the intensity is the prominent feature. The first three features used were average, minimum, and maximum intensity. The fourth feature is the size of a blob. Although each EX-ANs blob was small, some could be bigger than usual because they are adjacent and form a cluster. The size is the number of pixels in a blob.

#### Locations

**Fig 8:**
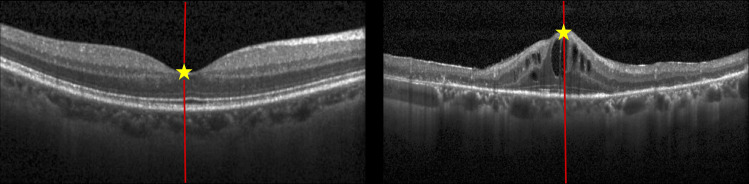
Illustrations of distances used as the 5^th^ to 7^th^ features (f5-f7) from a blob (orange circle) to the fovea.

From observations, EX-ANs emerge close to the fovea and lie within ILM and RPE layers. Therefore, we measured how far they are from the fovea and how deep they are under the ILM and RPE layers. Fig 8 illustrates the distances used as the fifth to seventh features.

**Fig 9:**
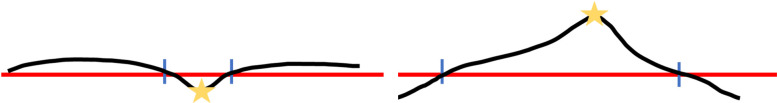
Examples of the fovea locations (stars) a typical case (left) and an abnormal case (right).

The explanation of how the fovea is located and the formulas for these distances are as follows. The fovea appears as a dip near the layer’s center in a typical symmetrical wing-shaped ILM layer. However, anomalies such as cysts or hemorrhages may result in the fovea being displaced upward, giving the ILM layer a mountain-like form. Fig 9 illustrates OCT images of normal and abnormal cases and their respective foveae. We conducted calculations based on the IML (I) and its fitted straight line (L) to determine the fovea point. The fovea is positioned at the intersection points of C and L, with the absolute distance in Euclidean geometry between this fovea point and *L* being minimized. The pseudo algorithm, FoveaDetection, employed in our study is outlined as follows:

Input: OCT image Img

Algorithm FoveaDetection(Img)

I = getIML(Img)

L = findlinearFitting(I)

{p1, p2} = findIntersections(I, L)

F = findFarthestPoint(I, L, p1, p2)

Output: a fovea point F

The algorithm requires the following functions.

getIML(Img): takes the image Img as an input and returns a set of points on the ILM

findlinearFitting(I): takes a set of points I and returns a set of points L of a linear fitting line of I

findIntersections(I, L): takes sets of points I and L, and returns two points on I and L of which the distance between them is less than *ϵ*. In our work *ϵ* = 10^−4^ is used in the calculation. When more than two points meet this requirement, it returns two points closest to the middle of I.

findFarthestPoint(I, L, p1, p2): takes a set of points I and L and points p1 and p2 and returns a point F on I between p1 and p2 of which the absolute distance between F to L is maximum.

**Fig 10:**
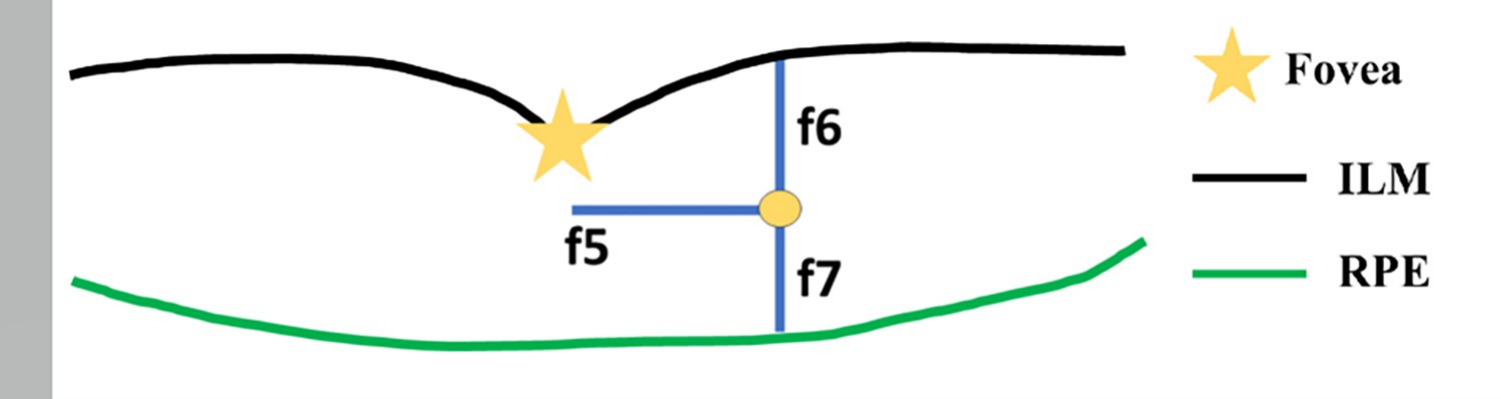
Illustrations of fovea detection procedure. The typical case (left) and the abnormal case (right) with ILMs (black lines), fitted lines (red lines), intersection points (blue vertical lines), and fovea (star).

Fig 10 illustrates the fovea detected in the normal and the diseased cases.

Given (x_b_, y_b_) the coordinate of the center of the blob, (x_f_, y_f_) the coordinate of the fovea point, *I* a set of points on the ILM boundary, *R* the set of points on the RPE boundary, and a function *getY*(*arg*,*x*) the *y* coordinate of the argument curve *arg* at *x*, the fifth feature is the difference in x between the blob and the fovea. The sixth feature was the y-distance between the blob center and the ILM curve. The seventh was the y-distance between the blob center and the RPE curve. The 5^th^-7^th^ distance features’ formulas are mathematically defined in Eqs (2)–(4).

Given (x_b_, y_b_) as the coordinates of the center of the blob (x_f_, y_f_) as the coordinates of the fovea point, *I* as a set of points on the ILM boundary, *R* as the set of points on the RPE boundary, and a function *getY(P*, *x)* representing the paired *y* coordinate of the input *x* in a set of points *P* representing a curve, the fifth feature is the difference in *x* between the blob and the fovea. The sixth feature represents the y-difference between the blob’s center and the ILM curve, while the seventh feature denotes the y-difference between the blob center and the RPE curve. The formulas for these 5^th^-7^th^ distance features are mathematically defined in Eqs (2)–(4):

$${Dist}_{Fov}={|x}_{b}-x_{f}|$$ (2)

$${Dist}_{ILM}={|y}_{b}-getY(I,x_{b})|,$$ (3)

$${Dist}_{RPE}={|y}_{b}-getY(R,x_{b})|,$$ (4)

#### A black shadow marker

**Fig 11:**
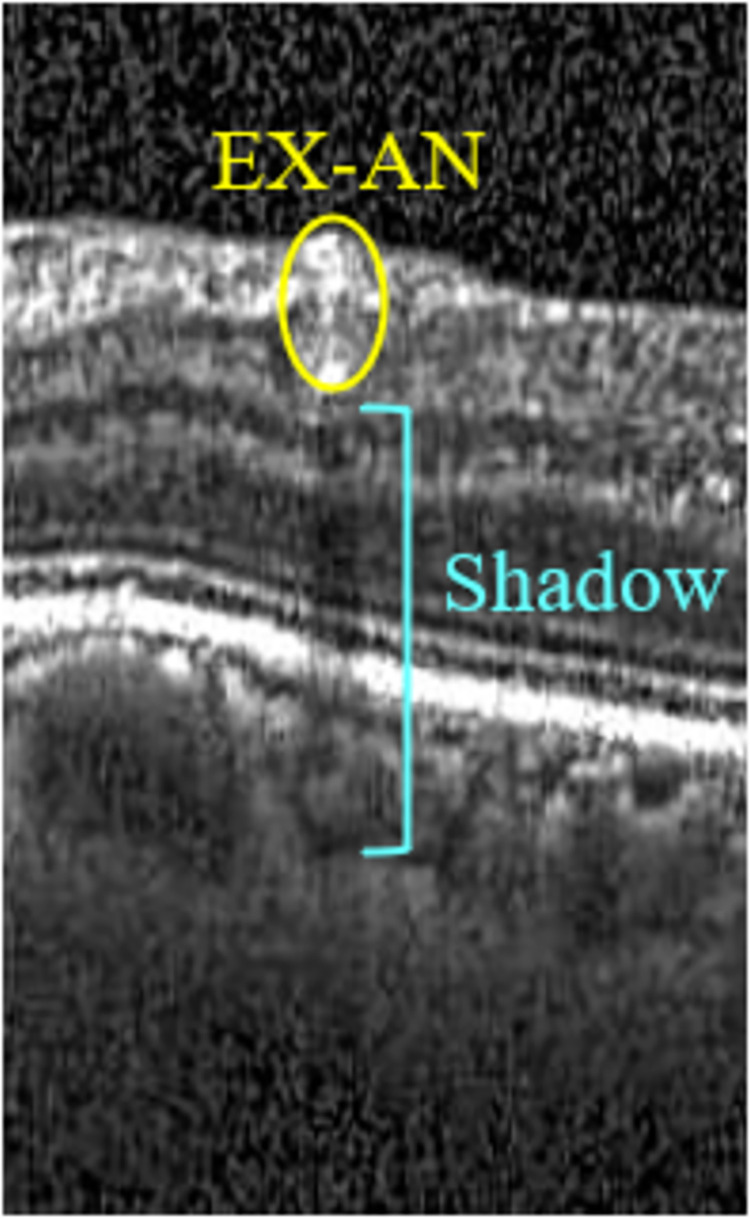
Illustration of a black shadow marker under the EX-AN.

EX-ANs blocked the penetration of the infrared light ray from OCT, resulting in thin black shadow lines below them. Fig 11 shows an OCT image depicting such shadows.

We identified the shadow marker as the dark, slender region beneath the EX-AN blob, modeling it as a thin rectangle. Since the shadow appears notably darker than its neighboring areas, we defined two additional rectangles of the exact dimensions along the sides of the tested region to represent the left and right neighboring regions. We then compared the intensities between these regions for shadow detection.

**Fig 12:**
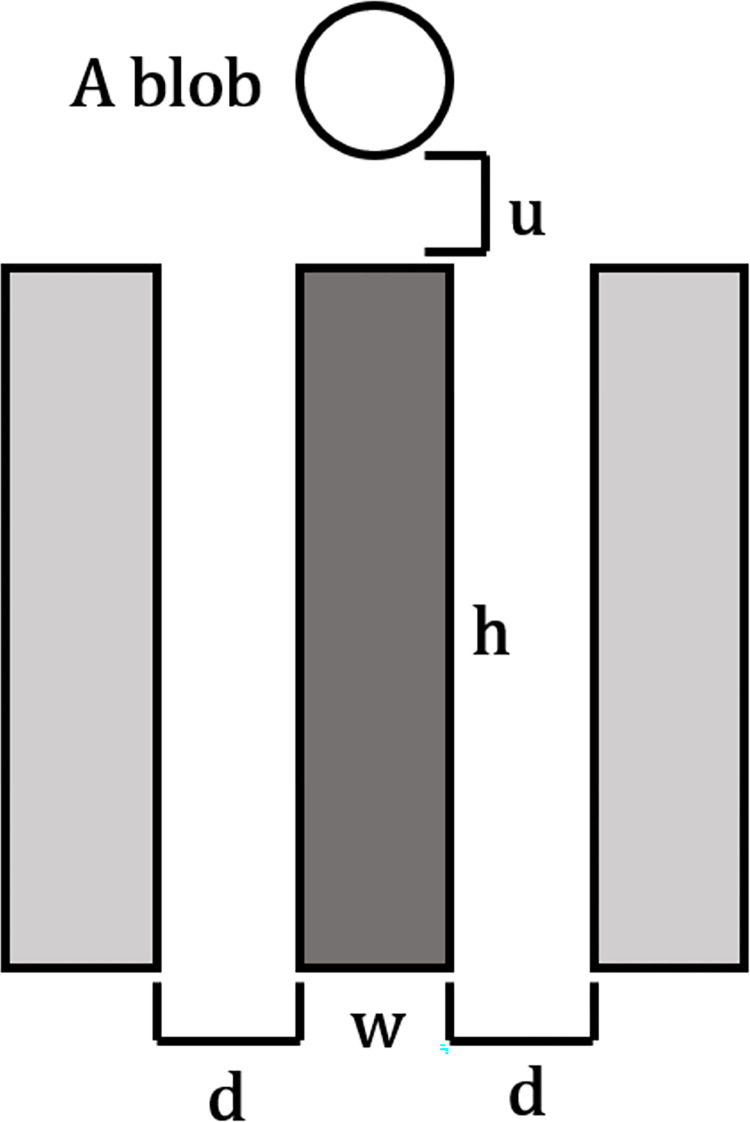
A model depicting the left (l), middle(m), and right(r) rectangular regions under the blob.

In Fig 12, we depicted three thin rectangular regions beneath a blob. A test rectangular region (*m*), with dimensions *w×h*, was positioned u pixels below the blob and occupies the middle position. The left (*l*) and right (*r*) rectangular regions, also having the exact dimensions and aligning parallel to the middle region, were situated *d* pixels away from the middle one.

In our experiment, we configured parameters *w*, *h*, *u*, and *d* to correspond to the blob’s width, triple the blob’s height, one-half the blob’s height, and a constant value of five pixels, respectively. These distances are measured in pixels.

The eighth feature is the differences in the average intensity of pixels between the middle and the left (*Idiff*_*l*_). The ninth feature is the average intensity of pixels between the middle and right (*Idiff*_*r*_). The calculations are in Eqs (2) and (3).

$$Idiff_{l}=|{\bar{I}}_{m}-{\bar{I}}_{l}|$$ (5)

and

$$Idiff_{r}=|{\bar{I}}_{m}-{\bar{I}}_{r}|$$ (6)

where ${\bar{I}}_{m},{\bar{I}}_{l},{\bar{I}}_{r}$ are the average intensities of pixels in the middle, left, and right rectangles, respectively.

High values of these differences indicate that the tested region is likely a shadow.

### EX-AN blob classifying using HRF features based bagged tree ensemble

**Fig 13:**
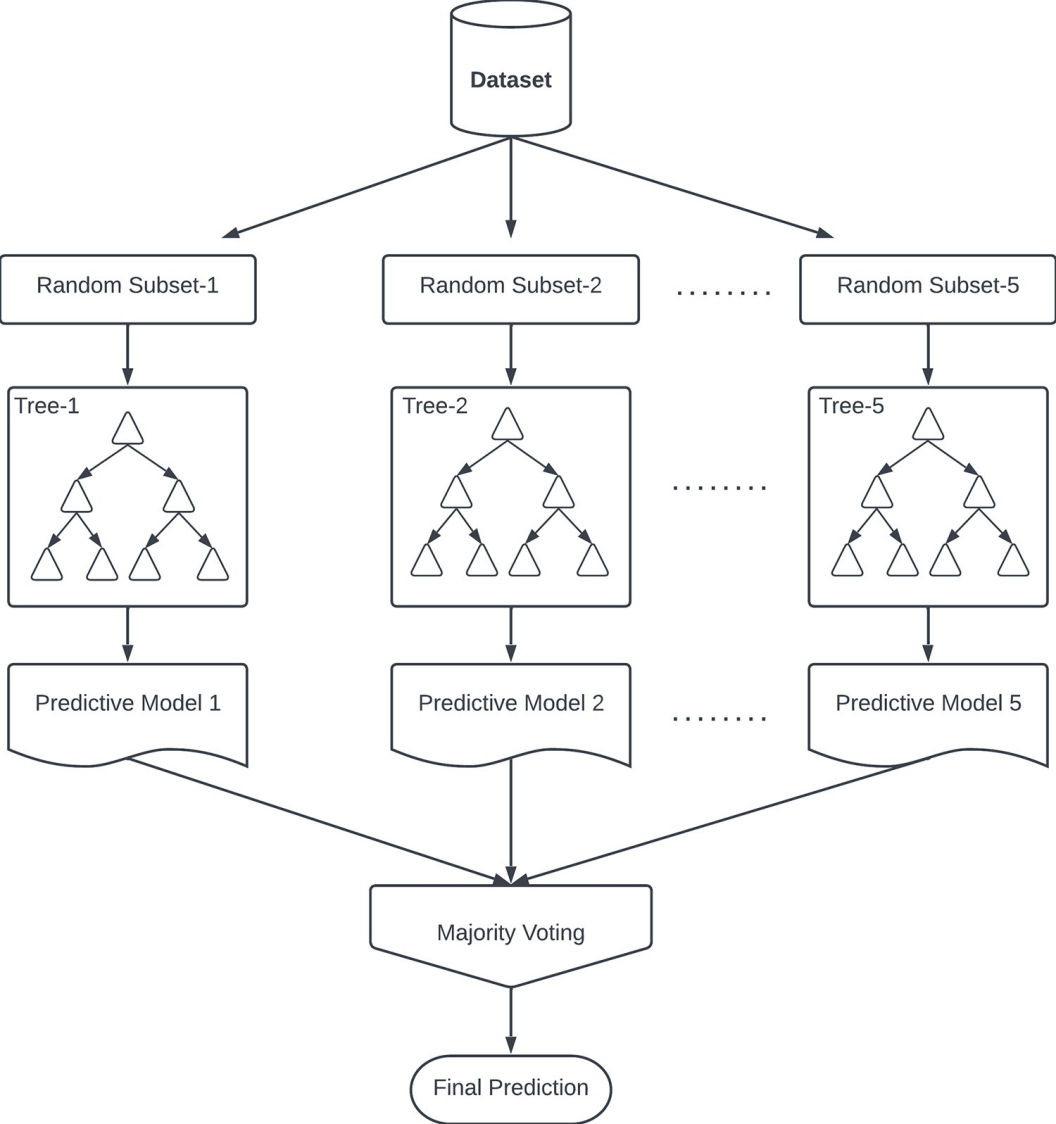
Processes in the bagged tree ensemble classification in our experiment.

We chose the bagged tree ensemble classifier [39] for our complex, high-variance, and low-bias feature data to mitigate overfitting in single models. The bagged tree ensemble builds multiple decision trees on different training data subsets, averting overfitting by aggregating diverse predictions and introducing randomness. Ensemble methods combine weak learners to create a strong learner. This ensemble approach enhances the model’s generalization ability across diverse data, offering improved performance. Fig 13 depicts the processes of the bagged tree ensemble classifier. The sub-samples were created from the input feature data. Each sample was then classified using an individual classifier to produce a predictive model.

Four folds trained the classifier during each iteration, while the fifth served as the test set. We ensured representative mixes of EX-AN and candidate blobs in each fold to prevent selection bias, preserving class distribution and statistical characteristics. Dataset randomization before partitioning helped minimize systematic biases. In addition, we maintained a strict boundary between sets to prevent inadvertent learning during training. The algorithm could not test set data for parameter updates or predictions, preserving independence. With a complete dataset, the risk of data leakage through accidental insertion during cross-validation was inherently minimized. This approach preserved training and test set independence, mitigating data leakage risks and enhancing the reliability of our five-fold cross-validation. The combined measures bolstered the validity of our classification model’s performance evaluation.

## Datasets and evaluation schemes

### Datasets

We employed a publicly available dataset consisting of 80 OCT B-scan images featuring hyperreflective foci, as documented by Kermany et al. [40]. Out of this compilation, 67 images exhibited EX-AN blobs. Expert annotators manually identified and marked all EX-AN blobs within the images to serve as ground truths. The images in the dataset were in JPEG format, with varying dimensions—the width ranging from 495 to 768 pixels and the height from 230 to 447 pixels.

We created training and testing sets to obtain the area of interest. The training set contained a total of 40 images. Due to our computer’s GPU, only 300 patches of dimension 224×224 could be randomly extracted from each image, determined by the smallest image dimension of 495×230.

There was a total of 12,000 patches in the training set. Ninety percent of each training set was used for training, and the remaining was used for validation. Well-trained observers interpreted the ground truth of the area of interest selection. The U-Net++ model was trained on each training set end-to-end using a computer with an Intel Core i7 CPU and an NVIDIA GeForce GTX 1070 Ti GPU. The training was performed for 50 epochs with a four-batch size and an initial learning rate of 0.0001. The RMSprop optimizer was used to reduce the learning rate adaptively. For binary classification problems, the binary cross-entropy loss function was widely used in training neural networks. Subsequently, dice loss was introduced to optimize the overlap between the ground truth and the predicted pixels for the segmentation problems. Then, we considered combining the dice loss in the overall loss function, resulting in a slight reduction in the loss curve. The loss function was based on a sum of the binary cross-entropy loss and the dice loss. The mathematical expression of the loss function (L) is as follows.

$$L_{BCE}=-(y\;\log\left(\hat{p}\right))+(1-y)log(1-\hat{p})$$ (7)

$$L_{D}=1-\frac{2y\hat{p}}{y+\hat{p}}$$ (8)

$$L=L_{BCE}+L_{D},$$ (9)

where *y* is the ground truth defined in [0, 1]and the $\hat{p}$ is sigmoid activation defined in [0, 1].

All programs were implemented using MATLAB R2021b. Five-fold cross-validation was used to train and test the classification model. Blobs that intersect with the ground truth more than 30% of their size were classified as positive.

### Evaluations

The segmentation performance was quantitatively evaluated using precision, recall, and F1-measure (or Dice Similarity Coefficient). Given the number of blobs that are true positives (TP), true negatives (TN), false positives (FP), and false negatives (FN), the formulas of these metrics are described in Eqs 2–4.


$$Precision=\frac{TP}{TP+FP}$$ (10)

$$Recall=\frac{TP}{TP+FN}$$ (11)

$$F1-measure=2\times \frac{Precision\times Recall}{Precision+Recall}$$ (12)

Remark that the area between the ILM and RPE layers is based on matched pixels, while the evaluation of blob segmentation is based on the matched blobs.

## Experiments and results

The following sub-sections compare performances of the UNET++ method used for area-of-interest segmentation and a bagged tree ensemble used for blob classification against comparative methods.

### The area between ILM and RPE layers

**Fig 14:**
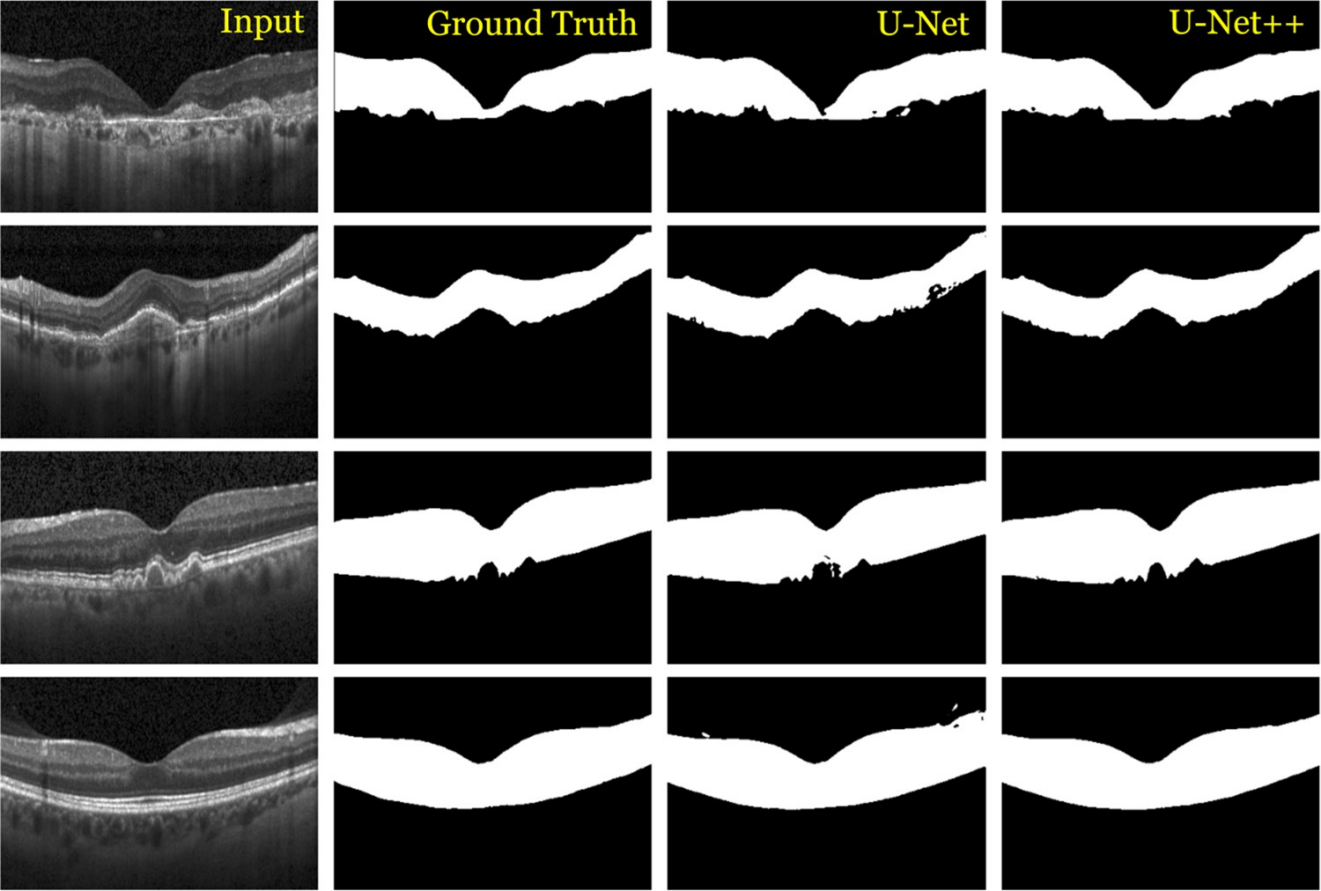
Examples of input (first column) and areas between ILM and RPE layers of ground truth (second column) and U-Net (third columns) and U-Net++ (fourth column) of four OCT cases.

The performance of U-Net++ was compared with U-Net. Fig 14 shows examples of the region of interest segmentation results from U-Net and U-Net++, together with the corresponding ground truths. Table 1 compares the numerical results of U-Net and U-Net++. U-Net++ outperforms U-Net, yielding high precision, recall, and F1-measure of 96.7, 98.7, and 97.7, respectively.

**Table 1:** Performance comparison of the area between ILM and RPE layers.

Methods	Metric		
	Precision	Recall	F1-Measure
U-Net	96.5	98.2	97.3
U-Net++ (our method)	96.7	98.7	**97.7**

### Blob segmentation

The blob’s regions were segmented by the AT method. EX-AN blobs were filtered from these blobs using the machine learning approach based on HRF features. We compare the performance of the proposed method with two comparative methods. The first is binarized thresholding with watershed segmentation (BT-WS) [32]. The second is AT combined with the shadow tracking (ST) method [33] (AT-ST).

**Fig 15:**
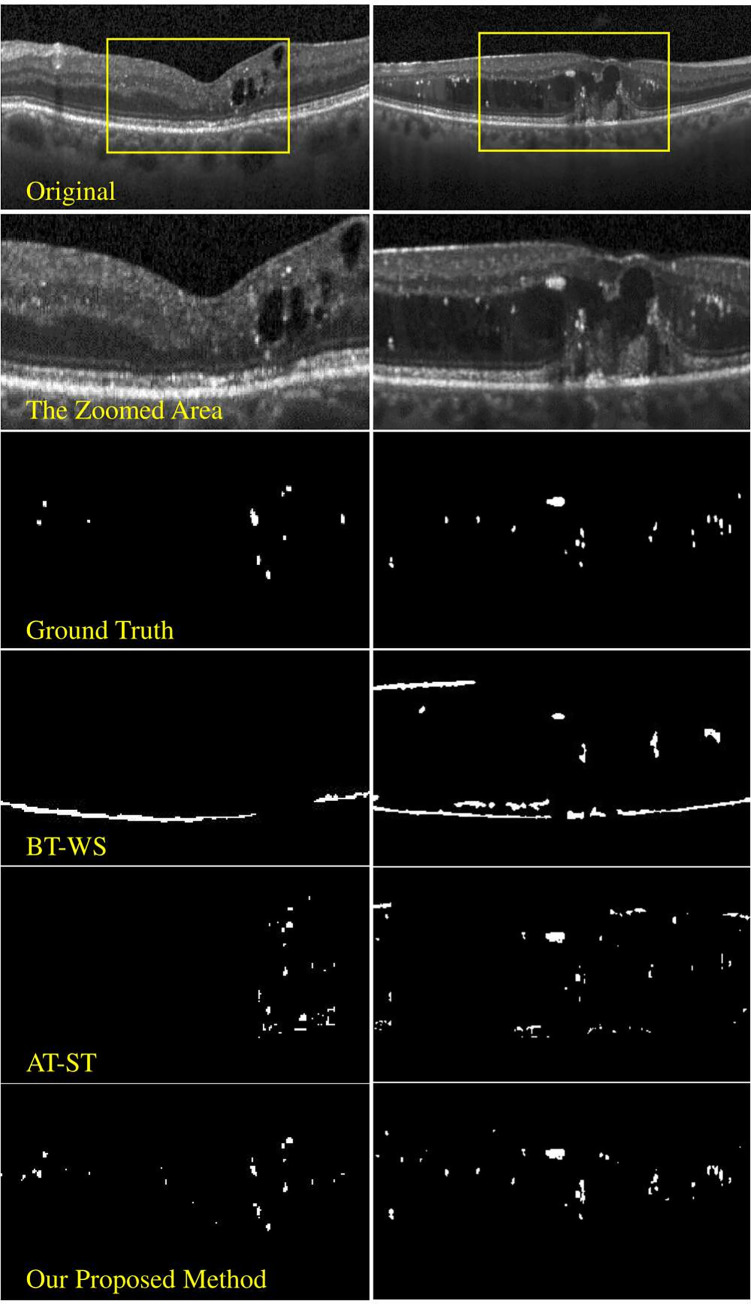
Examples of results from two different OCT images shown in the left and right columns.

Fig 15 shows qualitative results from two selected cases on the left and right columns. Our proposed method could segment EX-AN blobs more accurately compared to other methods. The BT-WS method incorrectly detected bright reflections on the regions of ILM and RPE.

The AT-ST method performs the poorest as it only considers blobs with shadows. The AT-ST method could not detect EX-ANs that have unclear shadows or did not have any shadows. Furthermore, some other pathologies, such as cysts, which appeared as black regions, wrongly cause AT-ST to detect blobs above these areas as shadows.

Table 2 depicts two performance comparisons. First is the performance of blob classifiers and their best models for EX-AN blob selection from all segmented blobs. Second, is the performance comparison of EX-AN segmentation from our approach and BT-WS and AT-ST.

**Table 2:** Comparison of our EX-AN segmentation method against comparative methods: BT-WS and AT-ST, and performance comparison of our used bagged tree ensemble classifier method against other classifiers and models.

EX-AN Segmentation Method			Performance (%)		
Blob Segmentation	Classifiers	Best Models	Precision	Recall	F1-measure
AT (our work)	Decision Tree	Medium Tree	81.4	84.3	82.8
	Discriminant Analysis	Quadratic Discriminant	61.7	**98.5**	75.9
	Logistic Regression	Logistic Regression	73.8	76.5	75.1
	Naïve Bayes	Kernel Naïve Bayes	72.3	88.1	79.5
	Support Vector Machine	Quadratic SVM	84.7	88.1	86.4
	Nearest Neighbor	Weighted KNN	80.5	86.8	83.6
	Neural Network	Narrow Neural Network	85.9	84.6	85.2
	Kernel Approximation	Logistic Regression Kernel	73.5	75.2	74.3
	**Ensemble** (our work)	**Bagged Trees** (our work)	**87.9**	86.1	**87.0**
BT-WS [36]			5.1	28.1	8.7
AT-ST [37]			2.7	27.0	4.9

The discussions of the findings and analysis of the results are provided in the Discussion section.

## Discussions

Among tested classifiers, the quadratic discriminant analysis yielded the highest recall rate of 98.5% but suffered the lowest precision rate of 61.7%. The bagged trees ensemble classifier outperformed the other classifiers on recall and F1-measure rates. It yielded the highest recall and F1-measures rates of 87.9 and 87.0%.

BT-WS and AT-ST performed poorly, with an average F1 measure of less than 10%. AT-ST performed the worst. Our proposed method remarkably outperformed the BT-WS method in the precision value by 82.8% and the recall value by 58.0%. Generally, our method’s F1 measure was ten times better than BT-WS’s.

Our method also significantly outperformed the AT-ST method in precision and recall by 85.2% and 59.1%, respectively. For the F1-measure, our proposed method is 82.1% or nearly 18 times higher than AT-ST.

Our ensemble bagged tree approach performed better than the BT-WS method [36] because our method utilized a more comprehensive set of features, while BT-WS used only an intensity feature. In addition, the watershed segmentation used in the BT-WS method for outlining their boundaries was unsuitable for low-quality and noisy OCT images. This finding agreed with the study of Yu et al. [41] that the watershed segmentation was prone to false segmentation on noisy data. Additionally, BT-WS finalized EX-AN blobs from candidate blobs using square or rectangle shapes. This decision was inefficient because it didn’t account for the various shapes collectively formed by the EX-AN blobs.

In the case of the AT-ST approach [37], they relied solely on shadows, which were determined based on the summation of the column pixels’ intensities. Thus, when a non-EX-AN recited on a cist area, which appeared black, it was incorrectly detected as an EX-AN. Moreover, because the EX-ANs were very small, their shadows often did not appear clearly. As a result, it was not surprising that their approach demonstrated the least effective performance.

**Fig 16:**
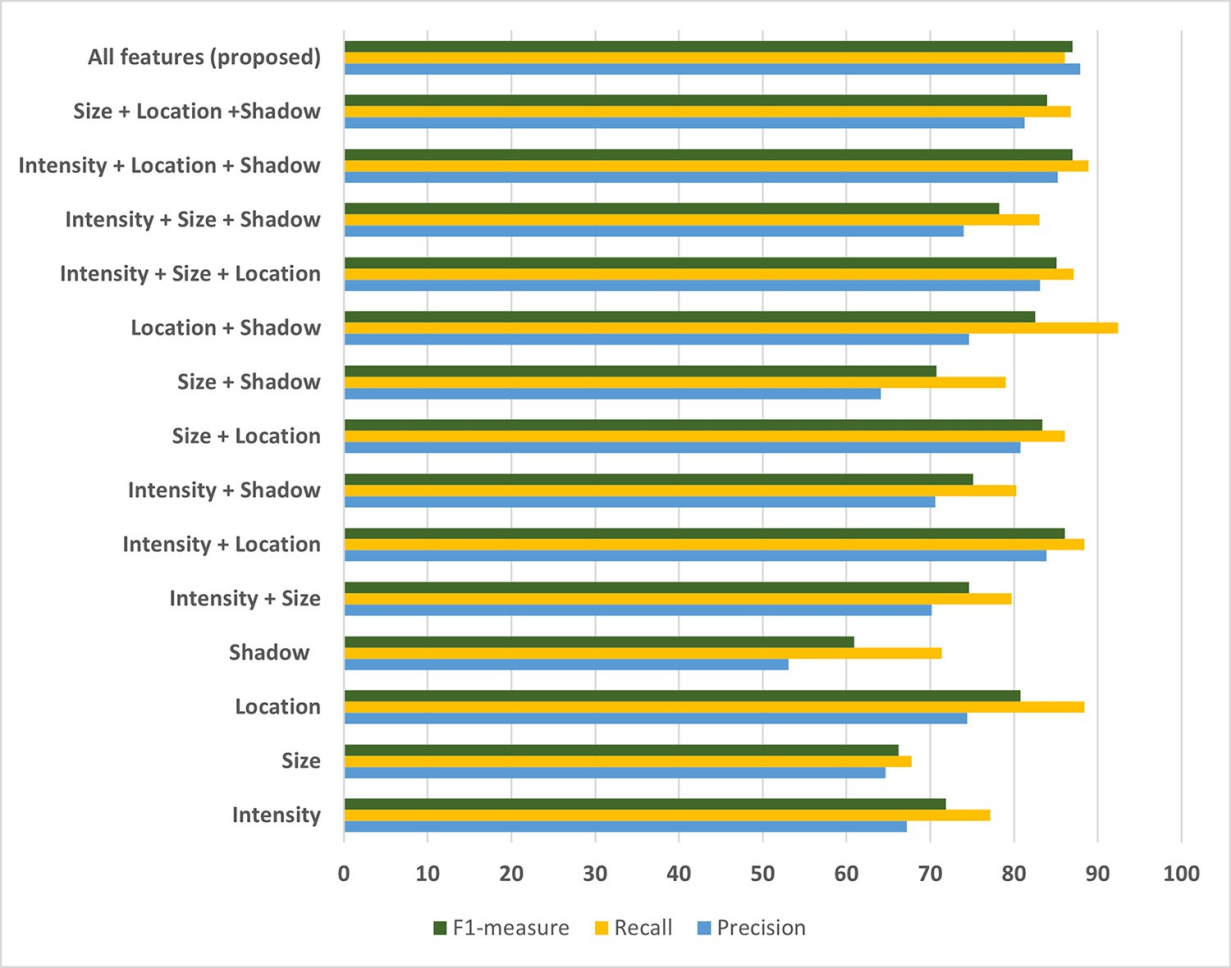
Performance comparison based on feature groups and their combinations.

Further analysis of how well each feature group and their combinations performed to the algorithm was depicted in Fig 16.

When comparing the performances of individual features, we observed that location, intensity, size, and shadow exhibit F1 scores in descending order. Blobs identified on hyper-reflective bands along the ILM and RPE lines may be mistakenly classified as EX-ANs due to their similar intensity range, leading to decreased precision. We could filter out certain irrelevant blobs by considering location features, enhancing overall precision.

Size plays a moderate role in our analysis, with most EX-ANs typically appearing as small blobs and being effectively detected. However, in severe cases, these blobs may form clusters of varying sizes, negatively impacting recall values. Furthermore, the size factor introduces the possibility of noise being mistaken for EX-AN, resulting in lower precision.

The shadow feature emerges as the least effective in correctly identifying EX-AN blobs. Weak shadows in small blobs can result in suboptimal observations. Additionally, dark non-shadow regions beneath EX-AN blobs may occasionally be misclassified as shadows.

As we combine more features, the F1 score increases. Single models yielded F1 scores ranging from 60.9 to 80.8, while double and triple-combined models yielded F1 scores ranging from 70.8 to 83.4 and 78.2 to 86.8, respectively. The combined model featuring all features attained the highest F1 score of 87.0. Notably, its precision significantly surpassed that of all other combined feature groups. Combining all features complemented features from each group, ensuring that the detected blobs exhibit the characteristics of EX-ANs.

**Fig 17:**
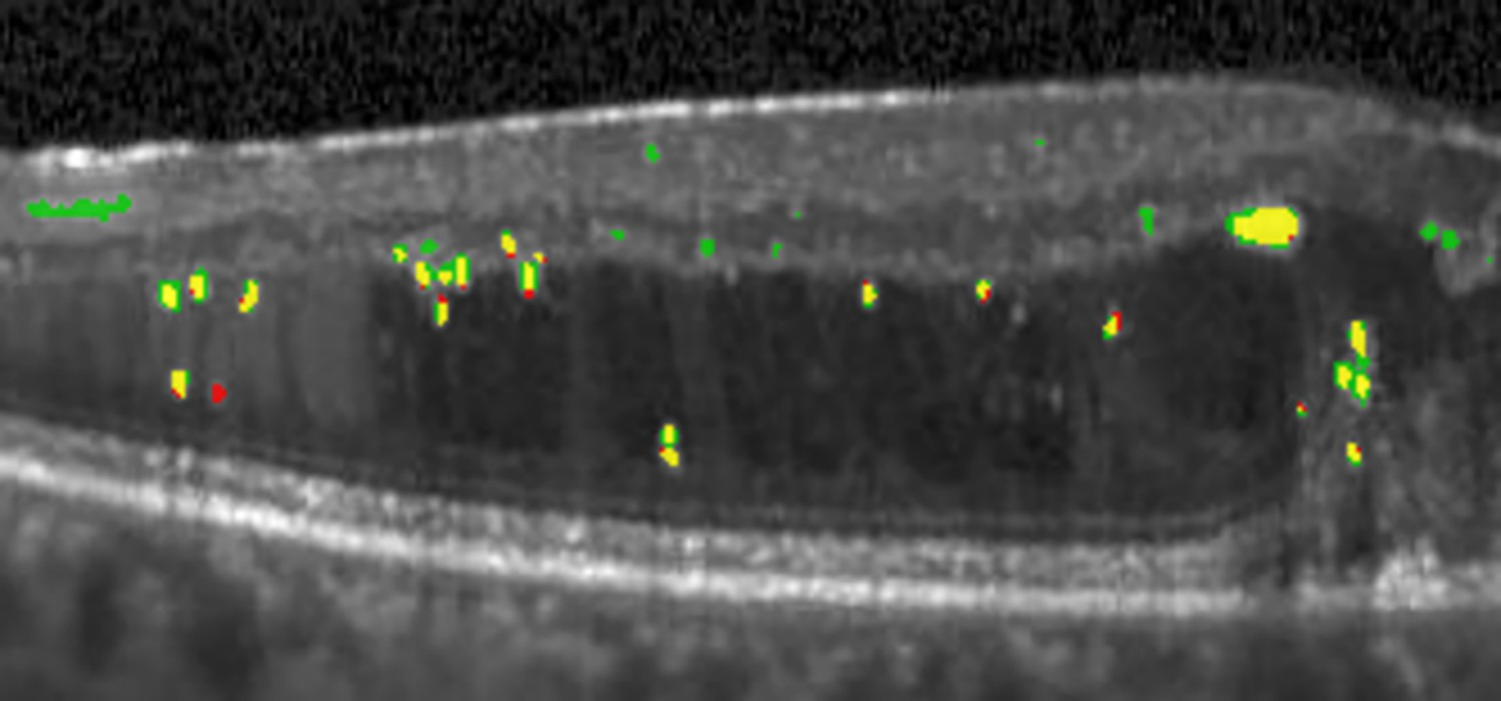
Illustration of the correct blobs (yellow), missing blobs (red), and irrelevant blobs (green).

Next is the error analysis. Fig 17 shows an example of our results’ correct, missing, and irrelevant blobs in an image. The following are error analyses from our results. Our proposed method could not retrieve some of the EX-AN blobs because of the imperfect prediction of the classifier. The missing blobs were usually not as bright as other blobs. The irrelevant blobs originated from various sources, such as noise or small tissue with unusually bright intensity.

Moreover, these occurrences occasionally occurred over tissues or cysts with non-uniform intensity. These areas potentially led to misinterpretation as shadows. Consequently, the blobs were mistakenly classified as EX-AN.

Incorrect cases due to bright, thick ILM/RPE layers can be solved by improving the detection algorithm. Retrieving irrelevant blobs could be solved by adding more cases with blobs beside/inside ILM and RPE or adding more features to the model. The problem of undetected blobs due to similar intensity values to their surrounding can be solved by improving the pre-processing process (contrast enhancement) and by enhancing the segmentation process.

## Conclusion

Optical Coherence Tomography (OCT) is an advanced imaging technique that utilizes infrared light waves to generate detailed, cross-sectional views of tissue structures. This technique has shown great promise in diagnosing and screening ocular diseases, especially diabetic retinopathy (DR). Within retinal fundus images and OCT scans, two indicators of DR anomalies, exudates (EX) and aneurysms (AN), can be observed. In OCT images, these indicators appear as hyperreflective (HRF) blobs, crucial biomarkers for early DR detection. Our study employed deep learning, image processing, and machine learning techniques to segment the EX-AN blobs. Our approach was evaluated on a dataset of 80 OCT images with ground truths. We utilized the deep learning U-Net++ model to detect the region of interest. We employed an adaptive thresholding method and a bagged tree ensemble based on the HRF features of the blobs to segment the EX-AN blobs. Our method outperformed two comparative methods (BT-WS and AT-ST) in performance evaluation. Specifically, our proposed method achieved a superior F1 measure compared to the state-of-the-art BT-WS and AT-ST methods by 78.3% and 82.1%, respectively. However, it is essential to note that the performance of our method depends on the quality and normality of the images. It may not perform well on low-contrast images or if the features used in this work are rendered inefficient by the presence of a disease. Finding a better contrast improvement method and more features that can help overcome these limitations will be a task in the future.

## References

[1] Huang D, Swanson EA, Lin CP, Schuman JS, Stinson WG, Chang W, et al. Optical coherence tomography. Science. 1991; 254(5035):1178–1181. doi: 10.1126/science.1957169 1957169 PMC4638169

[2] Swanson EA, Izatt JA, Hee MR, Huang D, Lin CP, Schuman JS, et al. In vivo retinal imaging by optical coherence tomography. Optics Letters. 1993; 18(21):1864–1866. doi: 10.1364/ol.18.001864 19829430

[3] Hee MR, Izatt JA, Swanson EA, Huang D, Schuman JS, Lin CP, et al. Optical coherence tomography of the human retina. Archives of Ophthalmology. 1995; 113(3):325–332. doi: 10.1001/archopht.1995.01100030081025 7887846

[4] Puliafito CA, Hee MR, Lin CP, Reichel E, Schuman JS, Duker JS, et al. Imaging of macular diseases with optical coherence tomography. Ophthalmology. 1995; 102(2):217–229. doi: 10.1016/s0161-6420(95)31032-9 7862410

[5] Neely KA, Quillen DA, Schachat AP, Gardner TW, Blankenship GW. Diabetic Retinopathy. Medical Clinics of North America. 1998; 82(4):847–876. doi: 10.1016/s0025-7125(05)70027-4 9706124

[6] Ogurtsova K, Guariguata L, Barengo NC, Ruiz PL, Sacre JW, Karuranga S, et al. IDF Diabetes Atlas: Global estimates of undiagnosed diabetes in adults for 2021. Diabetes Research and Clinical Practice. 2022; 183:109118, ISSN 0168-8227. doi: 10.1016/j.diabres.2021.109118 34883189

[7] International Diabetes Federation (IDF) IDF Diabetes Atlas 10th Edition. ISBN: 978-2-930229-98-0, www.diabetesatlas.org (accessed 4 September 2022).

[8] Teo ZL, Than YC, Yu M, Chee ML, Rim TH, Cheung N, et al. Global prevalence of diabetic retinopathy and projection of burden through 2045. American Academy of Ophthalmology. 2021; 128(11):1580–1591. 10.1016/j.ophtha.2021.04.027 33940045

[9] Gella L, Raman R, Rani PK, Sharma T. Spectral domain optical coherence tomography characteristics in diabetic retinopathy. Oman Journal of Ophthalmology. 2014; 7(3):126–129. doi: 10.4103/0974-620X.142594 25378876 PMC4220398

[10] Bolz M, Schmidt-Erfurth U, Deak G, Mylonas G, Kriechbaum K, Scholda C. Optical coherence tomographic hyperreflective foci. Ophthalmology. 2009; 116(5):914–920. 19410950 10.1016/j.ophtha.2008.12.039

[11] Fragiotta S, Abdolrahimzadeh S, Dolz-Marco R, Sakurada Y, Gal-Or O, Scuderi G. Significance of hyperreflective foci as an optical coherence tomography biomarker in retinal diseases: Characterization and Clinical Implications. Hindawi Journal of Ophthalmology. 2021; Article ID 6096017. 10.1155/2021/6096017 PMC8709761 34956669

[12] Mokhtari M, Ghasemi Kamasi Z, Rabbani H. Automatic detection of hyperreflective foci in optical coherence tomography B-scans using morphological component analysis. In Proceedings in the 39th Annual International Conference of the IEEE Engineering in Medicine and Biology Society. 2017; pp. 1497–1500. 10.1109/EMBC.2017.8037119 29060163

[13] Chen Z, Li D, Shen H, Mo Y, Wei H, Ouyang P. Automated retinal layer segmentation in OCT images of age-related macular degeneration. IET Image Processing. 2019; 13(11):1824–1834.

[14] Dodo BI, Li Y, Kaba D, Liu X. Retinal layer segmentation in optical coherence tomography. IEEE Access. 2019; 7:152388–152398.

[15] Okuwobi IP, Fan W, Yu C, Yuan S, Liu Q, Zhang Y, et al. Automated segmentation of hyperreflective foci in spectral domain optical coherence tomography with diabetic retinopathy. Journal of Medical Imaging. 2018; 5(1):1–16. doi: 10.1117/1.JMI.5.1.014002 29430477 PMC5800482

[16] Okuwobi IP, Ji Z, Fan W, Yuan S, Bekalo L, Chen Q. Automated quantification of hyperreflective foci in SD-OCT with diabetic retinopathy. IEEE Journal of Biomedical and Health Informatics. 2020; 24(4):1125–1136. doi: 10.1109/JBHI.2019.2929842 31329137

[17] Mukherjee S, Silva TD, Grisso P, Wiley H, Tiarnan DLK, Thavikulwat AT, et al. Retinal layer segmentation in optical coherence tomography (OCT) using a 3D deep-convolutional regression network for patients with age-related macular degeneration. Biomedical Optics Express. 2022; 13(6):3195–3210. doi: 10.1364/BOE.450193 35781941 PMC9208604

[18] Khaing TT, Okamoto T, Ye C, Mannan MA, Yokouchi H, Nakano K, et al. ChoroidNET: a dense dilated U-Net model for choroid layer and vessel segmentation in optical coherence tomography images. IEEE Access. 2021; 9:150951–150965.

[19] Ronneberger O<, Fischer P, Brox T. U-Net: Convolutional networks for biomedical image segmentation. In Proceedings in International Conference on Medical Image Computing and Computer-Assisted Intervention (MICCAI), 2015; pp. 234–241.

[20] Siddique N, Paheding S, Elkin CP, Devabhaktuni V. U-Net and its variants for medical image segmentation: theory and applications. IEEE Access. 2021; 9:82031–82057.

[21] Zyuzin V, Chumarnaya T. Comparison of Unet architectures for segmentation of the left ventricle endocardial border on two-dimensional ultrasound images. In Proceedings in Ural Symposium on Biomedical Engineering, Radioelectronics and Information Technology (USBEREIT). 2019; pp. 110–113.

[22] Zhou Z, Siddiquee MMR, Tajbakhsh N, Liang J. U-Net++: A nested U-Net architecture for medical image segmentation. in Deep Learning in Medical Image Analysis and Multimodal Learning for Clinical Decision Support. Cham, Switzerland: Springer, 2018; 3–11. 10.1007/978-3-030-00889-5_1 PMC7329239 32613207

[23] Kugelman J, Allman J, Read SA, Vincent SJ. Tong J, Kalloniatis, et al. A comparison of deep learning U-Net architectures for posterior segment OCT retinal layer segmentation. Scientific Reports. 2022; 12:14888. doi: 10.1038/s41598-022-18646-2 36050364 PMC9437058

[24] Yojana K and Thillai Rani L. OCT layer segmentation using U-Net semantic segmentation and RESNET34 encoder-decoder. Measurement: Sensors. 2023; 29:100817.

[25] Shelhamer E, Long J, Darrell T. Fully convolutional networks for semantic segmentation. IEEE Transactions on Pattern Analysis and Machine Intelligence, 2017; 39(4):640–651. doi: 10.1109/TPAMI.2016.2572683 27244717

[26] Katona M, Kovacs A, Varga L, Grosz T, Dombi J, Degi R, et al. Automatic detection and characterization of biomarkers in OCT images. In Proceedings in International Conference Image Analysis and Recognition. 2018; pp. 706–714.

[27] Schlegl T, Bogunovic H, Klimscha S, Seebock P, Sadeghipour A, Gerendas B, et al. Fully automated segmentation of hyperreflective foci in optical coherence tomography images. In Proceedings in IEEE Conference Computer Vision and Pattern Recognition. 2018; Article No. 1131308.

[28] Yu C, Xie S, Niu S, Ji Z, Fan W, Yuan S, et al. Hyper-reflective foci segmentation in SD-OCT retinal images with diabetic retinopathy using deep convolutional neural networks. Medical Physics, 2019; 46(10):4502–4519. doi: 10.1002/mp.13728 31315159

[29] Varga L, Kovacs A, Grosz T, Thury G, Hadarits F, Degi R, et al. Automatic segmentation of hyperreflective foci in OCT images. Computer Methods and Programs in Biomedicine. 2019; 178:91–103. doi: 10.1016/j.cmpb.2019.06.019 31416566

[30] Xie S, Okuwobi IP, Li M, Zhang Y, Yuan S, Chen Q. Fast and automated hyperreflective foci segmentation based on image enhancement and improved 3D U-Net in SD-OCT volumes with diabetic retinopathy. Translational Vision Science & Technology. 2020; 9(2): Article 21. doi: 10.1167/tvst.9.2.21 32818082 PMC7396192

[31] Yao C, Wang M, Zhu W, Huang H, Shi F, Chen Z, et al. Joint segmentation of multi-class hyper-reflective foci in retinal optical coherence tomography images. IEEE Transactions on Biomedical Engineering, 2022; 69(4):1349–1358. doi: 10.1109/TBME.2021.3115552 34570700

[32] Wei J, Yu S, Du Y, Kun L, Yupeng X. Automatic Segmentation of Hyperreflective Foci in OCT Images Based on Lightweight DBR Network. Journal of Digital Imaging. 2023. doi: 10.1007/s10278-023-00786-0 36749455 PMC10287852

[33] Schmidt MF, Christensen JL, Dahl VA, Toosy A, Petzold A, Hanson JV, et al. Automated detection of hyperreflective foci in the outer nuclear layer of the retina. Acta Ophthalmologica. 2022 101(2): 200–206. doi: 10.1111/aos.15237 36073938

[34] Niu S, Yu C, Chen Q, Yuan S, Lin J, Fan W, et al. Multimodality analysis of Hyper-reflective Foci and Hard Exudates in Patients with Diabetic Retinopathy. Scientific Reports. 2017; 7:1568. doi: 10.1038/s41598-017-01733-0 28484225 PMC5431476

[35] Szymkowski M, Saeed E, Saeed K, Mariak Z. A Simple Algorithm for Hard Exudate Detection in Diabetic Retinopathy Using Spectral-Domain Optical Coherence Tomography In: Gavrilova M., Chang J., Thalmann N., Hitzer E., Ishikawa H. (eds) Advances in Computer Graphics. CGI 2019. Lecture Notes in Computer Science(), vol. 11542. Springer, Cham.

[36] Patil A, Chakravorty C. Detection of hard exudate using retinal optical coherence tomography (OCT) images. Global Transitions Proceedings. 2021; 2:566–570.

[37] Singh M, Gupta V, Singh PK, Gupta R, Kumar B, Alenezi F, et al. Automatic detection of hard exudates shadow region within retinal layers of OCT images. Hindawi Mathematical Problems in Engineering. 2022; Article ID 7128547.

[38] Midena E, Torresin T, Velotta E, Pilotto E, Parrozzani R, Frizziero L. OCT Hyperreflective Retinal Foci in Diabetic Retinopathy: A Semi-Automatic Detection Comparative Study. Frontiers in Immunology, 2021; 12, 613051. doi: 10.3389/fimmu.2021.613051 33968016 PMC8100046

[39] Breiman L. Bagging Bagging predictors. Machine Learning. 1996; 24: 123–140.

[40] Kermany DS, Goldbaum M, Cai W, Valentim CC, Liang H, Baxter SL, et al. Identifying medical diagnosis and treatable diseases by image-based deep learning. Cell, 2018; 172(5):1122–1131. 29474911 10.1016/j.cell.2018.02.010

[41] Yu Y, Wang C, Fu Q, Koe R, Huang F, Yang B, et al, Techniques and Challenges of Image Segmentation: A Review, Electronics, 2023, 12(5), 1199.

